# The survival of murine hepatitis virus (a surrogate of SARS-CoV-2) on conventional packaging materials under cold chain conditions

**DOI:** 10.3389/fpubh.2023.1319828

**Published:** 2023-12-05

**Authors:** Tiancheng Xie, Jiaxue Yang, Chubin Fang, Jing Zhang, Hua Lin, Yalan Zhu, Tian Tang, Chuan Wang

**Affiliations:** ^1^West China School of Public Health and West China Fourth Hospital, Sichuan University, Chengdu, China; ^2^West China-PUMC C.C. Chen Institute of Health, Sichuan University, Chengdu, China; ^3^Technology Center of Chengdu Customs, Chengdu, China

**Keywords:** murine hepatitis virus, cold chain, disinfection, SARS-CoV-2, packaging materials

## Abstract

**Introduction:**

The cold chain conditions have been suggested to facilitate long-distance transmission of SARS-CoV-2, but it is unclear how viable the virus is on cold chain packaging materials.

**Methods:**

This study used the MHV-JHM strain of murine hepatitis virus as a model organism to investigate the viability of SARS-CoV-2 on foam, plastic, cardboard, and wood sheets at different temperatures (−40°C, −20°C, and 4°C). In addition, the ability of peracetic acid and sodium hypochlorite to eliminate the MHV-JHM on plastic and cardboard sheets were also evaluated.

**Results:**

The results indicate that MHV-JHM can survive on foam, plastic, or cardboard sheets for up to 28 days at −40°C and −20°C, and up to 14 days on foam and plastic surfaces at 4°C. Although viral nucleic acids were still detectable after storing at 4°C for 28 days, the corresponding virus titer was below the limit of quantification (LOQ).

**Discussion:**

The study highlights that a positive nucleic acid test result may not indicate that the virus is still viable, and confirms that peracetic acid and sodium hypochlorite can effectively eliminate MHV-JHM on packaging materials under cold chain conditions.

## 1 Introduction

Since late 2019, the outbreak of severe acute respiratory syndrome coronavirus 2 (SARS-CoV-2) has caused more than 758 million confirmed cases and 6.85 million deaths worldwide (1). SARS-CoV-2 spreads primarily through respiratory transmission, but evidence shows that it can also spread through contaminated environmental surfaces (2–4). Multiple studies have shown that the coronavirus is capable of surviving on a variety of surfaces for varying durations (5, 6). Specifically, studies have revealed that SARS-CoV-2 can remain viable on food surfaces for up to 21 days when stored in refrigeration (4°C) or freezing temperatures (−10°C to −80°C) (7). These conditions are essential for preserving perishable food, but they also provide favorable conditions for the survival of coronavirus. Although several outbreaks of COVID-19 have been linked to imported cold chain food (8, 9), information regarding the viability of the causative agent, SARS-CoV-2, on cold chain packaging materials is limited.

As SARS-CoV-2 must be handled in a Biosafety Level 3 (BSL-3) laboratory (10), surrogate coronavirus such as the JHM strain of murine hepatitis virus (MHV-JHM) have been extensively used as model viruses for studying SARS-CoV-2. The merit of using such viruses for mimicking SARS-CoV-2 is that they can be manipulated under BSL-2 laboratory conditions, reducing the threshold and cost of study. Additionally, viruses like MHV-JHM share the same genus with SARS-CoV-2 which could offer insights relative to SARS-CoV-2 studies (11, 12). Except for MHV-JHM, bacteriophage phi 6, transmissible gastroenteritis virus (TGEM), and human coronavirus OC43 were also used as surrogates to investigate coronaviruses (13, 14). In the present study, we analyzed the viability of MHV-JHM under the cold chain conditions. Our tests were performed on four packaging materials (foam, plastic, cardboard, and wood sheets) under three temperatures of −40°C, −20°C, and 4°C. The viability of virus was confirmed by 50% tissue culture infectious dose (TCID_50_). Since nucleic acid-based methods remain the gold standard for confirming the presence of coronavirus (15), it is sometimes mistakenly believed that the identified SARS-CoV-2 nucleic acid fragment has the same infectious potential as the actual virus in the environment. Therefore, we are intrigued to investigate whether a digital polymerase chain reaction (dPCR) positive material eventually tests negative for virus viability.

In practice, surface disinfection of cross-border goods is crucial to prevent the spread of SARS-CoV-2 (16). Spraying liquid chemical disinfectants is the most commonly used method for object disinfection (17). This study also compared the effectiveness of two disinfectants (peracetic acid and sodium hypochlorite) on plastic and cardboard sheets (18, 19), providing a reference for disinfection measures under cold chain conditions.

## 2 Materials and methods

### 2.1 Viruses, cells and packaging materials

Mouse fibroblast L929 cells were obtained from Yaji Biotechnology Co., Ltd. (Shanghai, China) and cultured in modified DMEM media composed of Dulbecco’s modified Eagle’s medium (DMEM) supplemented with 10% fetal bovine serum (FBS) and 1% penicillin-streptomycin. The cells were incubated at 37°C with 5% CO_2_. MHV-JHM was obtained from the American Type Culture Collection (ATCC) (VR-765) and propagated in mouse fibroblast L929 cells. In Brief, a 25 cm^2^ flask containing the L929 monolayers were inoculated with 50 μL of MHV-JHM and 450 μL of modified DMEM media. Followed by incubation at 37°C with 5% CO_2_ for 60 min, 4.5 mL of modified DMEM media was added to the flask. The virus were allowed to grow in L929 monolayers at 37°C with 5% CO_2_ until more than 95% of the cells exhibited cytopathic effects (CPEs).

Four types of cold chain packaging materials, each with a thickness of 1 mm, were used in this study. These included plastic boxes made of polyethylene terephthalate (Xian Yuan Packaging Co., Ltd., Xiamen, China), foam boxes made of expandable polystyrene (Hezi Packaging Materials Co., Ltd., Hunan, China), cardboard boxes (Shengtai Group, Jiangsu, China), and paulownia wood (Zhongtian Wood Co., Ltd., Shenzhen, China).

### 2.2 Concentration and purification of MHV-JHM

To prepare the virus stocks, cell cultures were frozen at −80°C for at least 1 h and then thawed in a 37°C water bath to lyse the cells and release the virus. The lysate was then centrifuged at 986 × g for 10 min at 4°C to pellet the cell debris. The supernatant was collected and further clarified by centrifugation at 5,000 × g for 1 h at 4°C to remove impurities. Followed by loading over 1 mL of 30% sucrose solution (w/v) and centrifuged at 112,500 × g for 2.5 h at 4°C, the virus pellet was resuspended in 1.8 mL of modified DMEM media and aliquoted. The virus stocks were frozen at −80°C until use. The titer of the virus stocks were calculated according to Reed and Muench method (20).

### 2.3 Viability of coronavirus on cold chain packaging materials

**Figure 1:**
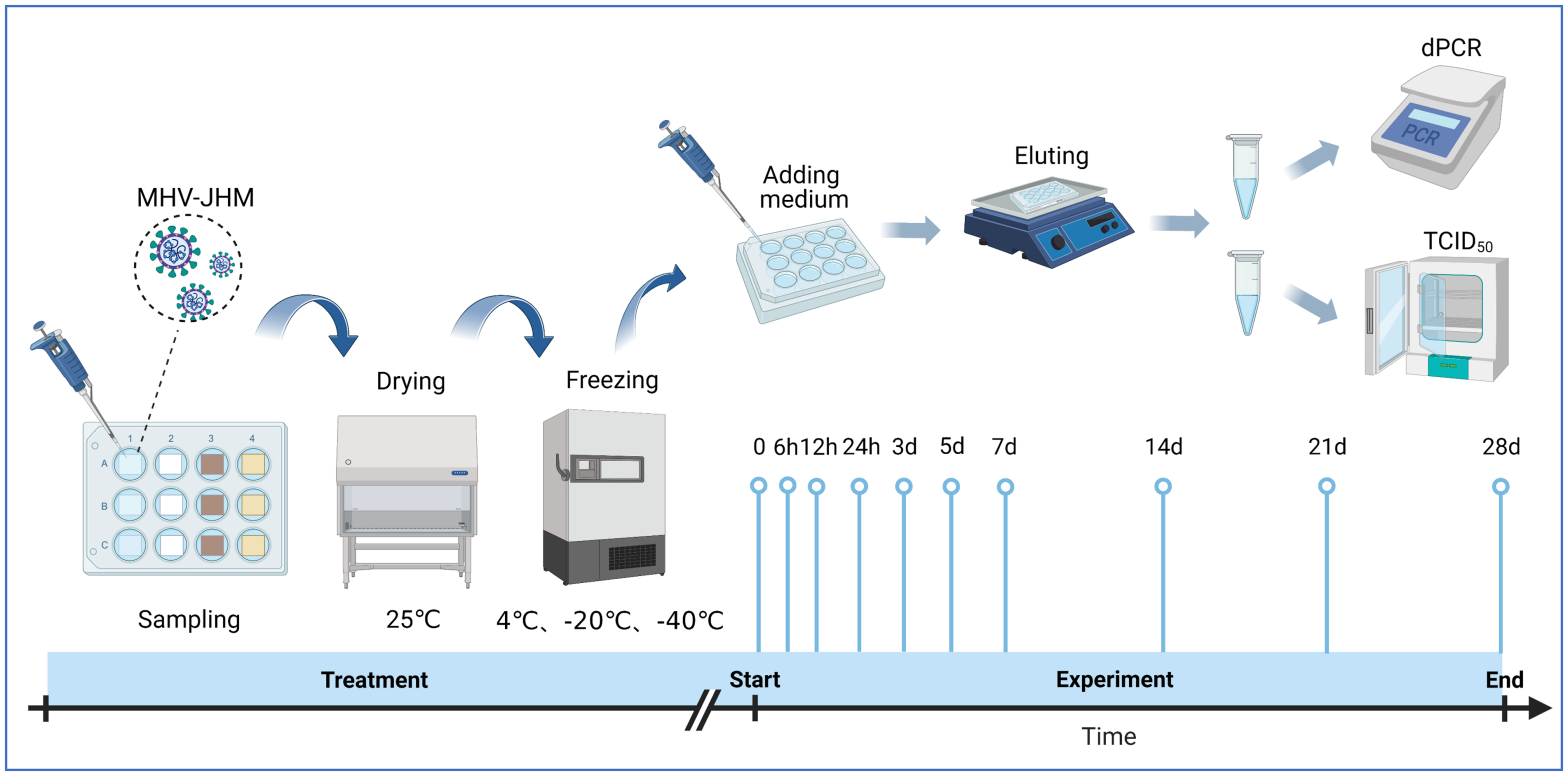
Experimental procedure for evaluating surface viability of MHV-JHM on cold chain packaging materials. Source: Created with BioRender.com.

To prepare the virus containing packaging materials, sheets of 1.4 cm × 1.4 cm were cut from the materials. Followed by sterilization with ethylene oxide and placed into a 12-well cell culture plate, 25 μL of MHV-JHM virus stocks were pipetted onto the surface of the packaging materials. The plate was left to dry at 25°C for 40 min in a biosafety cabinet. After drying, the samples were incubated at −40°C, −20°C, and 4°C for 0, 6, 12, 24 h, 3, 5, 7, 14, 21, and 28 days. At each time point, 500 μL of modified DMEM media was added to each well, and the plate was shaken at 30 rpm on ice for 10 min. The eluate was then collected, aliquoted, and frozen at −80°C for the subsequent TCID_50_ assay and dPCR analysis (Figure 1). The image of sample preparation is listed in Supplementary Figure S1.

### 2.4 Spraying disinfection experiment

To perform the spraying disinfection experiment, we utilized two types of disinfectants: 0.3% peracetic acid and 2000 mg/L sodium hypochlorite. The disinfectants were diluted with phosphate-buffered saline (PBS) to their recommended concentrations and were pre-chilled at either −20°C or 4°C (with added antifreeze: 28% NaCl). To ensure consistent and uniform spraying, the virus containing packaging materials were sprayed at a specific distance (20 cm) and angle (45°) using a spray bottle. After spraying, the samples were stored at −20°C or 4°C for 24 h. A control group was included where PBS was used instead of the disinfectants while maintaining all other procedures. We added 0.5% sodium thiosulfate to the virus elution to neutralize any residual disinfectant. The virus titer was calculated using the Reed and Muench method as before.

### 2.5 Quantitation of viral titers using a 50% tissue culture infectious dose assay

The L929 cells were seeded at a density of approximately 20,000 cells per well in 96-well plates and incubated for 16 h at 37°C with 5% CO_2_. The virus stock solution was serially diluted in modified DMEM media. Each dilution was then added to six wells of a 96-well plate containing L929 cell culture monolayers (six replicates per dilution). The plates were incubated for two to 5 days and observed daily to monitor the development of cytopathic effects (CPEs), and the virus titers calculated.

### 2.6 RNA extraction and digital polymerase chain reaction

To extract the viral RNA, we used either the Nucleic Acid Extraction and Purification Kit (Vazyme Co. Ltd., Nanjing, China) or the VNP-32P Automated Nucleic Acid Extraction System (Vazyme Co. Ltd., Nanjing, China) following the instructions provided by the manufacturer. We then performed a digital PCR (dPCR) using the High One-Step RT-dPCR Probe Super Mix (Cy5.5) kit (Sniper Co. Ltd., Suzhou, China). The dPCR mixture contained 3.5 μL of One-step RT-dPCR master mix probe buffer, 1 μL of enzyme mix, 1 μL each of the forward primer (5’-TACTAGGTTTGCGCCCGGTA-3′) and reverse primer (5′-CGCTGGTTGGAACTGCTTCT-3′), 1 μL of TaqMan probe (5′-ATCTGGTTCGCGGCCACAATCCCGT-3′), 2 μL of RNA template, and 12.5 μL of enzyme-free water. The dPCR assays were performed using a DQ24 digital PCR instrument (Sniper Co. Ltd., Suzhou, China). The specificity of the primers used in dPCR was confirmed by polymerase chain reaction (PCR) and quantitative polymerase chain reaction (qPCR) (Supplementary Figure S2). The reaction systems and protocols involved in PCR and qPCR are listed in Supplementary Table S1. The probe concentration was optimized and shown in Supplementary Figures S3, S4. The cycle threshold (Ct) value is a key parameter for determining the presence or absence of coronavirus. Therefore, a TaqMan probe-based quantitative polymerase chain reaction (qPCR) was employed to detect nucleic acid samples and determine their copy numbers by using a known copy number plasmid as a reference standard. This method was used to convert the number of nucleic acid copies into Ct (cycle threshold) values. The reaction components and steps of the TaqMan probe-based qPCR are listed in Supplementary Table S2.

### 2.7 Statistical analyses

The statistical analysis was conducted using R Studio (version 4.2.2). Normality of the variables was determined using the Shapiro–Wilk test. For variables with normal distribution, one-way analysis of variance (ANOVA) was used to assess differences between groups. In cases where the data did not follow a normal distribution, the Mann–Whitney *U* test or Kruskal–Wallis test was utilized. Statistical significance was set at a *p*-value of less than 0.05.

## 3 Results

### 3.1 The viability of MHV-JHM on cold chain packaging materials

**Figure 2:**
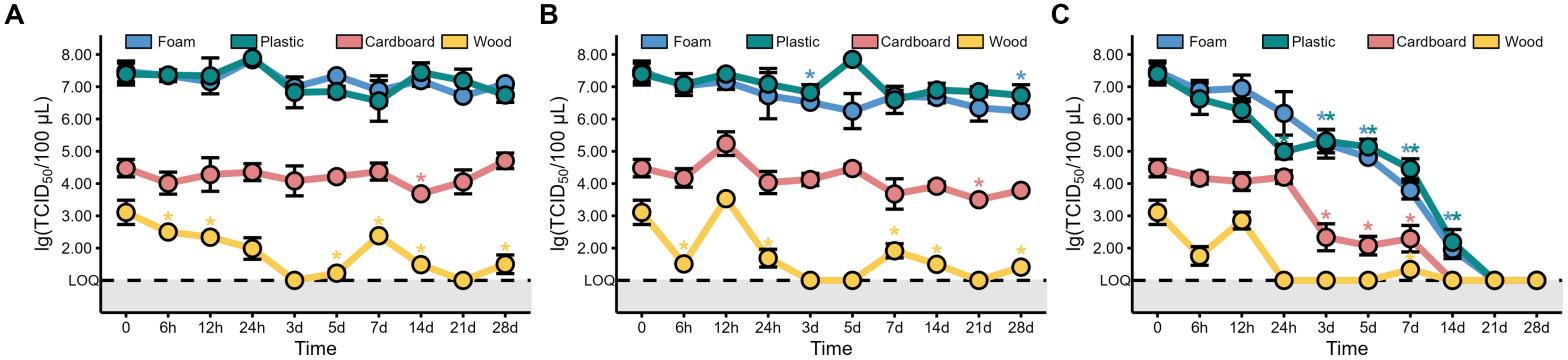
The titers of MHV-JHM on the foam, plastic, cardboard, and wood sheets at −40°C **(A)**, −20°C **(B)**, and 4°C **(C)**. LOQ means limit of quantification, and grey backgrounds indicate values below the limit of detection. All data are shown as the mean ± s.e. The virus titer on a packaging material at various sampling times was compared to the initial virus titer. A *p*-value less than 0.05 was considered statistically significant, and indicated by asterisks (*).

The TCID_50_ results showed that the viability of MHV-JHM decreased over time at 4°C on all packaging materials (Figure 2C). However, no significant decrease (*p* > 0.05) in MHV-JHM viability was observed after 24 h on foam and cardboard sheets. On plastic sheets, the titer of MHV-JHM decreased from 10^7.4^ to 10^5.3^ TCID_50_/100 μL after 24 h, while on wood sheets, it was below the limit of quantification (LOQ) within the same time period. After 3 days, the titers of MHV-JHM decreased by two orders of magnitude on foam, plastic, and cardboard sheets from 10^7.5^, 10^7.4^, and 10^4.5^ TCID_50_/100 μL to 10^5.2^, 10^5.3^, and 10^2.3^ TCID_50_/100 μL, respectively. From day 3 to day 7, the titers of MHV-JHM on foam and plastic sheets decreased by 1.4 and 0.8 log units, respectively. After 14 days, the titer of MHV-JHM on cardboard sheets was below the LOQ, and on day 21, the titers of MHV-JHM on foam and plastic sheets were also below the LOQ.

Interestingly, no significant difference (*p* > 0.05) was found in the titers of MHV-JHM on all packaging materials storing at −40°C and −20°C between day 0 and day 28, except the foam sheets at −20°C where the titer of MHV-JHM differed between day 0 and day 28 (Figures 2A,B). The titer of MHV-JHM on wood sheets was below the LOQ after 3 days and fluctuated thereafter at −40°C and −20°C.

**Figure 3:**
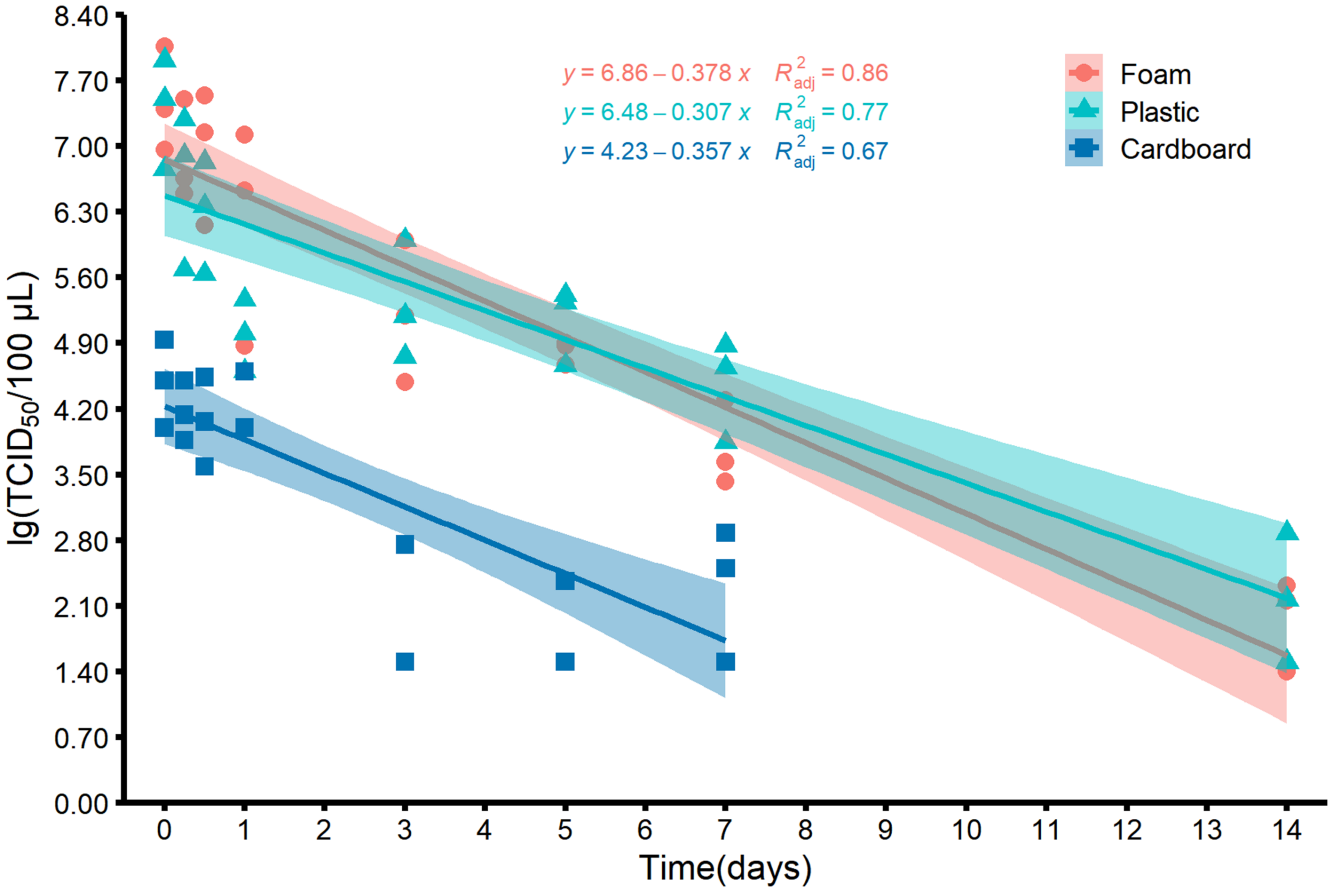
Scatter plots and the corresponding fitted lines showing the correlations between the titers of MHV-JHM on the surfaces of foam, plastic, and cardboard samples and time at 4°C. Smoothing curves based on linear model separately for each material are shown in different color with 95% confidence intervals. *R*^2^_adj_ means the adjusted determination coefficient.

Since low temperatures such as −40°C and −20°C barely change the viability of viruses over time, we decided to explore the relationship between virus titers and storage time at 4°C by using the maximum likelihood estimation algorithm. Figure 3 depicts a linear correlation between storage time and virus titers on foam, plastic, and cardboard sheets. The adjusted determination coefficient (*R*^2^_adj_) values were 0.86, 0.77, and 0.67, respectively, with corresponding slopes of −0.378, −0.307, and −0.357. The virus titer on plastic and cardboard sheets decreased significantly over time, with a reduction of 0.3–0.4 log units per day at 4°C. The titer of virus on cardboard sheets was only depicted for the first 7 days, because it fell below the LOQ after 14 days (Figure 3).

### 3.2 The nucleic acid levels of MHV-JHM on packaging materials over time

**Figure 4:**
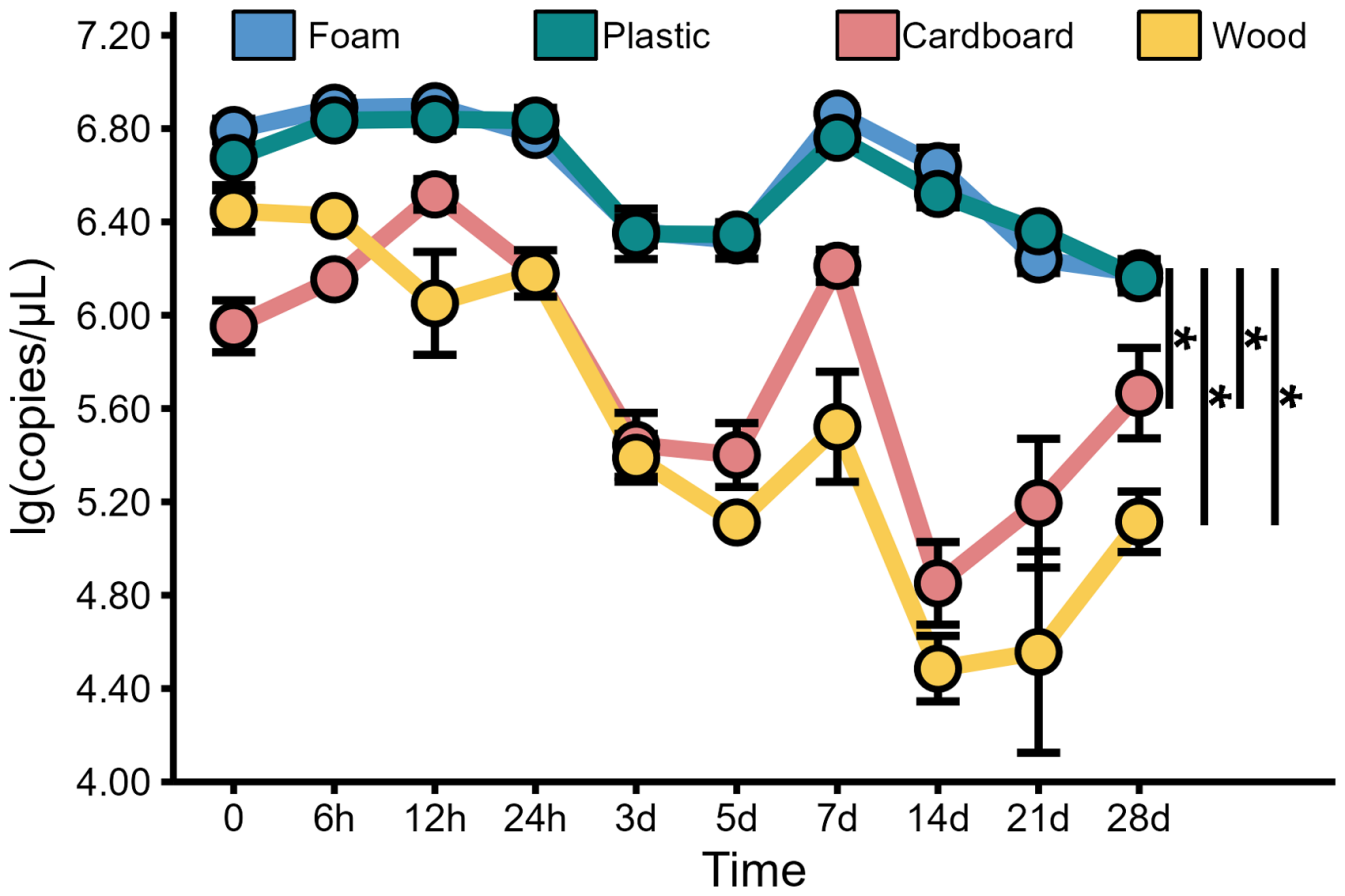
The viral copy numbers of MHV-JHM on the foam, plastic, cardboard, and wood sheets at 4°C. All data are shown as the mean ± s.e. A *p*-value less than 0.05 was considered statistically significant, and indicated by asterisks (*).

**Figure 5:**
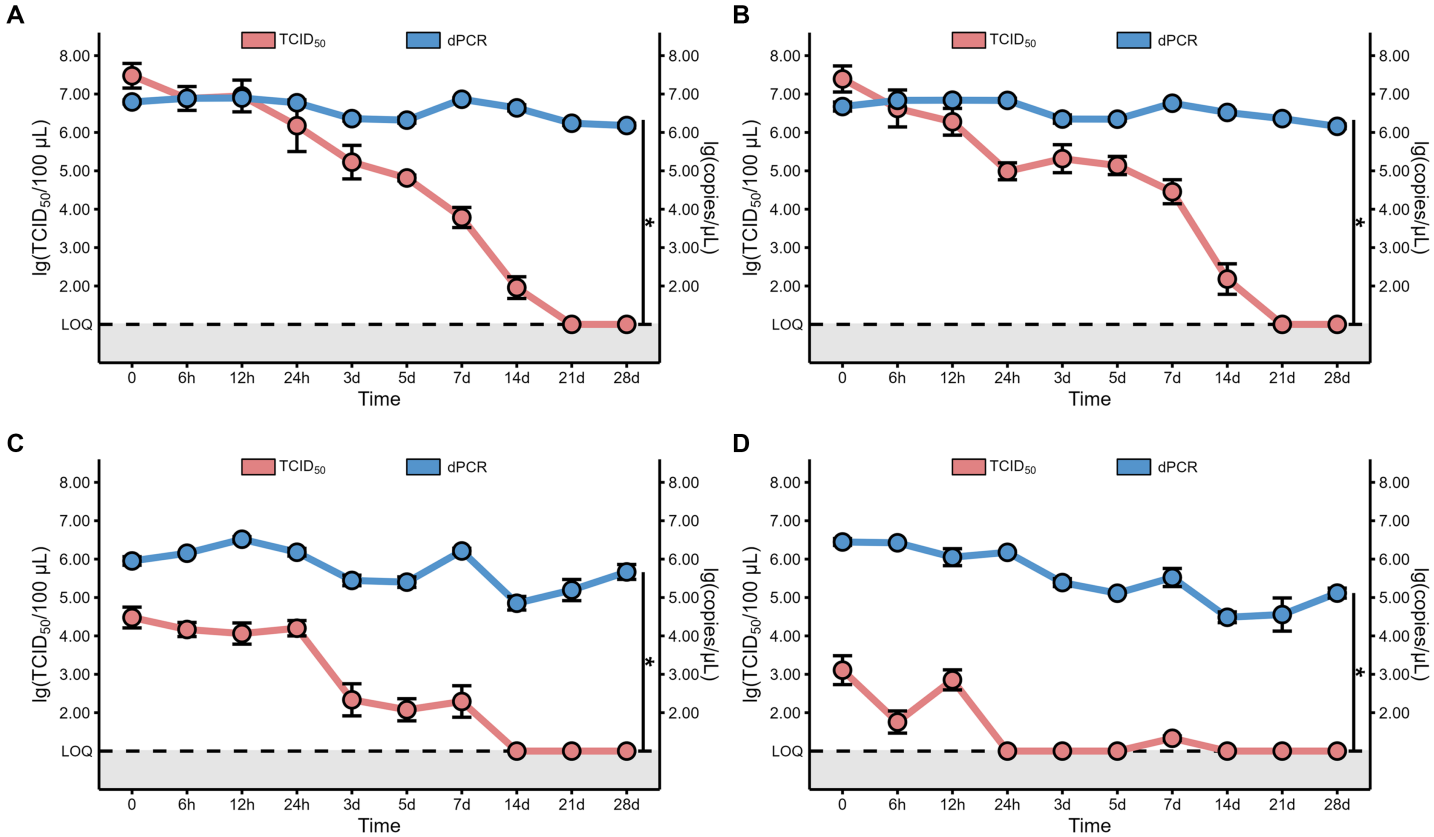
The comparison between the viral copy numbers and the virus viability of MHV-JHM on the foam **(A)**, plastic **(B)**, cardboard **(C)**, and wood sheets **(D)**, at 4°C. LOQ means limit of quantification, and grey backgrounds indicate values below the limit of detection. All data are shown as the mean ± s.e. A *p*-value less than 0.05 was considered statistically significant, and indicated by asterisks (*). The red line represents the virus viability, which was confirmed by 50% tissue culture infection dose (TCID50). The blue line represents the viral copy numbers, which was determined by digital polymerase chain reaction (dPCR).

In addition to the virus titer, we also determined the nucleic acid levels of MHV-JHM on different packaging materials at 4°C over time. Our findings revealed consistent high levels of MHV-JHM nucleic acid (10^4.5^ to 10^6.9^ copies/μL) on all packaging materials at 4°C (Figure 4). Over the course of 28 days, the nucleic acid levels of MHV-JHM on foam, plastic, cardboard, and wood sheets decreased from 10^6.8^, 10^6.7^, 10^5.9^, and 10^6.4^ copies/μL to 10^6.2^, 10^6.2^, 10^5.7^, and 10^5.1^ copies/μL, respectively. Significant differences in virus nucleic acid concentration were observed between foam and cardboard sheets, foam and wood sheets, plastic and cardboard sheets, and plastic and wood sheets (*p* < 0.05). Nucleic acid concentrations of MHV-JHM were higher in foam and plastic sheets compared to cardboard sheets (0.8 ± 0.4 log units) and wood sheets (1.1 ± 0.6 log units) at different time points (mean ± standard deviation) (Figure 5).

As the gold standard for SARS-CoV-2 confirmation, the nucleic acid (NA) test raises questions about the virus viability of samples testing positive. In China, a CT value below 35 confirms positivity, typically determined using the qPCR. We used digital PCR (dPCR) for its superior sensitivity, necessitating the conversion of dPCR results, the viral copy cumbers, into CT values to determine sample positivity. The sample subjected to qPCR analysis had a known copy number of viral nucleic acid which allowed for the estimation of the Ct value. The linear regression formula that converts the Ct value to copy number is *Y* = −3.466*X* + 39.70, where *Y* represents the Ct value while *X* represents copy number (Supplementary Figure S5). According to this formula, the Ct values for viral RNA on foam, plastic, cardboard, and wood sheets stored at 4°C on day 28 were consistently below 35, estimated to be 9.67, 9.67, 9.81, and 9.98, respectively.

### 3.3 The effects of spraying disinfection treatment on the removal of MHV-JHM

**Figure 6:**
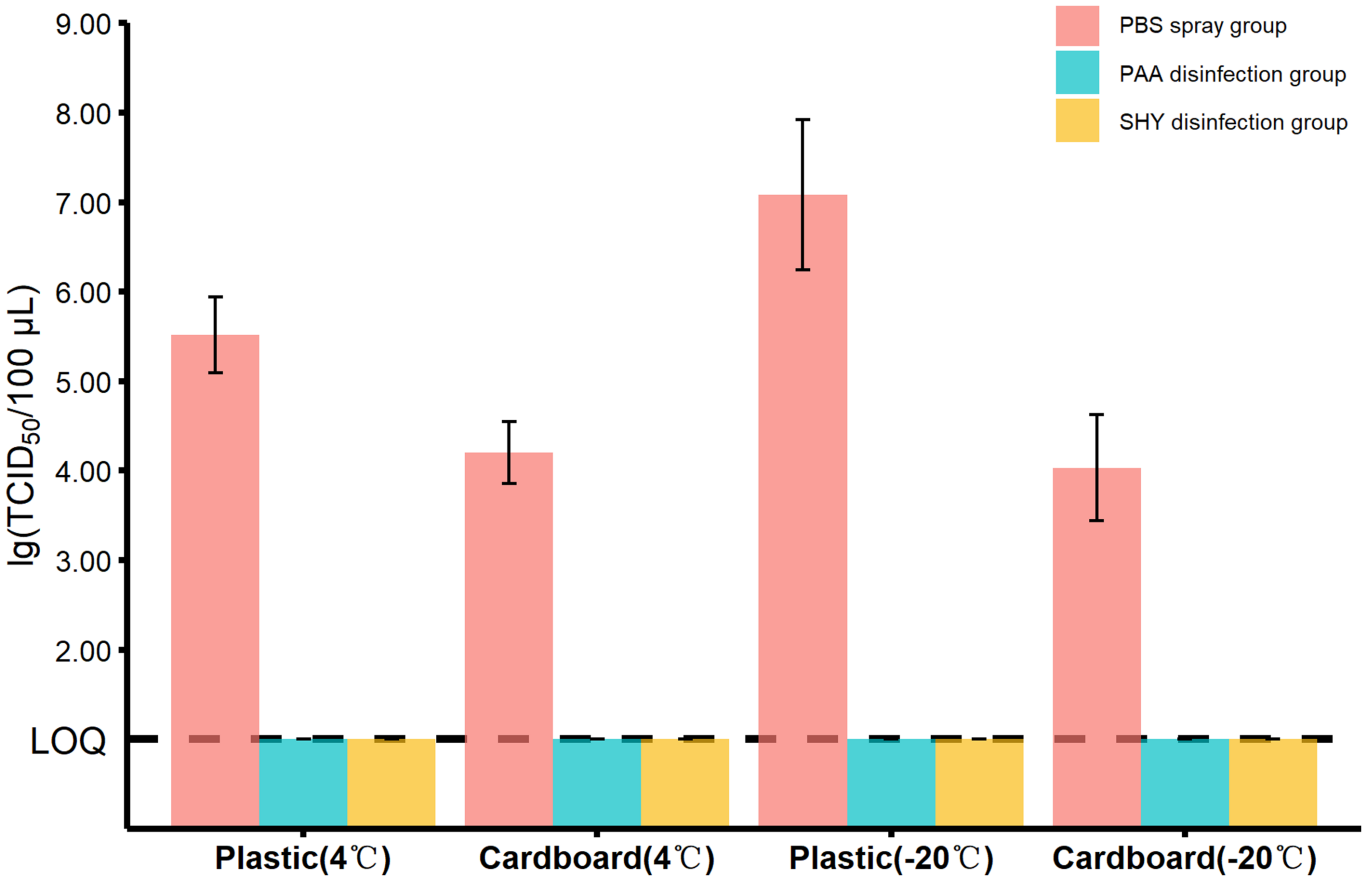
The titers of MHV-JHM in different groups on the plastic and cardboard sheets in spraying disinfection experiment. PBS means phosphate buffered saline. PAA means peracetic acid. SHY means sodium hypochlorite. LOQ means limit of quantification. All data are shown as the mean ± s.e.

As the titer of MHV-JHM presented a similar dynamic pattern at both −40°C and −20°C, we therefore use −20°C as a model temperature to investigate the effect of disinfectants on the viability of coronavirus. Additionally, we conducted a similar experiment at 4°C as a parallel control. The experiments were performed on plastic and cardboard sheets, since these materials are not just cheap but also easy to obtain. In the spraying disinfection experiment, the dosage of disinfectants been used was determined by weighing the packaging samples before and after spraying (Supplementary Table S3), which was 21.46 ± 7.20 g/m^2^ (mean ± standard deviation). As shown in Figure 6, the titer of virus on both materials were below the LOQ in the presence of peracetic acid or sodium hypochlorite at 4°C, with a killing rate of >99.99%. Similar results were also observed while incubation at −20°C. Notably, the plastic sheets had a higher titer of virus in the absence of any disinfectants, irrespective of the storing temperature (Figure 6).

## 4 Discussion

The persistence of airborne viruses on material surfaces poses a great threat to susceptible individuals. The viability of coronavirus on cold chain packaging materials stored at three different temperatures (−40°C, −20°C, and 4°C) were investigated using a SARS-CoV-2 surrogate, MHV-JHM. The results showed that MHV-JHM remained viable on foam, plastic, and cardboard sheets for the entire 28 days duration at both −40°C and −20°C. Additionally, the viability of MHV-JHM was maintained for up to 14 days at 4°C on foam and plastic surfaces, which is consistent with previous studies (21, 22). It has been suggested that environmental conditions are important factors affecting the viability of coronaviruses (5). Our study found that MHV-JHM was more stable on foam and plastic surfaces compared to wood and cardboard sheet surfaces at 4°C. We attribute this to the rapid evaporation of virus-containing microdroplets on the surface of porous materials (wood and cardboard sheets) which impairs the viability of MHV-JHM (23, 24), given that the drying condition is able to denature the viral protein, change the viral lipid, and finally, result in a lower titers of virus (25). In addition to water evaporation, other conditions such as pH and the concentration of salts also determined the “fate” of the virus (26). It is important to note that the copy number of MHV-JHM on wood sheet surfaces decreased dramatically over time. Perhaps, the rapid inactivation of virus on wood sheet surfaces accelerated the degradation of viral RNA. Despite adding an equal volume of MHV-JHM microdroplets to foam, plastic, cardboard, and wood sheet surfaces, the virus titer in the elution from wood or cardboard sheet samples were lower than those from foam and plastic sheet samples. This phenomenon prompt us to suspect that the risk of coronavirus infection through porous packaging materials, such as wood and cardboard sheets, might be lower than the risk of infection through impermeable packaging materials, such as foam or plastic sheets. Further evidence is required to substantiate this hypothesis.

In addition to investigating virus viability, we also examined the effectiveness of disinfectant sprays in eliminating coronavirus attached to packaging surfaces. While previous studies have focused on disinfection at temperatures ranging from 4°C to 68°C (27), little evidence exists regarding the effectiveness of sanitizers under cold chain conditions. Alcohol-based, quaternary ammonium, peroxide, and chlorine disinfectants are commonly used in the cold chain environment (18, 22). However, alcohol-based disinfectants may cause plastic materials to swell and harden, and the efficacy of quaternary ammonium disinfectants is reduced in materials with high water hardness or absorbency (28). Therefore, this study evaluated the efficacy of peracetic acid, a peroxide disinfectant, and sodium hypochlorite, a chlorine disinfectant, in eliminating MHV-JHM. Our data indicated that both disinfectants were effective in inactivating the viruses at −20°C or 4°C, achieving a kill rate of greater than 99.99%. As the virucidal activity of disinfectants against coronaviruses was typically determined by the carrier tests or the suspension tests (29), the present data provides valuable information regarding the practical application of disinfectants in direct spray form (30).

To associate the NA results with the viral viability, we utilized the dPCR technique, which provided highly reproducible and precise results with exceptional sensitivity (31). The dPCR results highlights the discovery that a positive nucleic acid (NA) test result may not indicate that the virus present in the sample is still alive and infectious, as has been previously reported (18, 32, 33). It was demonstrated that various coronaviruses share comparable environmental tolerance (27), our data obtained from MHV-JHM could gain further insights into the survival of other coronaviruses on the packaging materials at low temperature conditions, such as SARS-CoV-2.

It is important to show the limitations of the present study. Since the surrogate virus MHV-JHM was utilized instead of SARS-CoV-2, future studies should consider using the real SARS-CoV-2 to validate the present findings. Additionally, the viability of coronavirus on a wider range of cold-chain packaging materials should be investigated. Recent studies have shown that the viability of coronavirus is close related to several factors, such as relative humidity and pH (34), the exact impact of these cofactors should be addressed in the upcoming studies. In addition, we only assessed the efficacy of two disinfectants on the elimination of coronavirus, future study should explore more disinfectants and investigate their effectiveness in eliminating the SARS-CoV-2 virus on cold chain packaging materials.

## 5 Conclusion

In summary, this study found that the SARS-Cov-2 surrogate virus MHV-JHM could survive on foam, plastic, and cardboard surfaces for 28 days at −40°C and −20°C. It could also survive for up to 14 days on foam and plastic surfaces at 4°C. The risk of coronavirus infection through porous packaging materials, such as wood and cardboard sheets, might be lower. Additionally, we underscore that a positive nucleic acid (NA) test result may not indicate that the virus present in the sample is still alive and infectious. The MHV-JHM on packaging materials can be eliminated at cold chain conditions by using peracetic acid or sodium hypochlorite.

## Data availability statement

The original contributions presented in the study are included in the article/Supplementary material, further inquiries can be directed to the corresponding authors.

## Ethics statement

Ethical approval was not required for the studies on animals in accordance with the local legislation and institutional requirements because only commercially available established cell lines were used.

## Author contributions

TX: Writing – original draft, Writing – review & editing, Conceptualization, Data curation, Investigation, Methodology, Validation, Visualization. JY: Writing – review & editing, Conceptualization, Investigation. CF: Writing – review & editing, Conceptualization, Investigation, Methodology, Validation. JZ: Writing – review & editing, Project administration, Resources. HL: Writing – review & editing, Project administration, Resources. YZ: Writing – review & editing, Conceptualization. TT: Funding acquisition, Supervision, Writing – review & editing, Conceptualization, Project administration, Resources. CW: Funding acquisition, Supervision, Writing – review & editing, Conceptualization, Project administration, Resources.

## References

[1] World Health Organization. Global COVID-19 statistics. Available at: https://covid19.who.int/

[2] Lu R Zhao X Li J Niu P Yang B Wu H. Genomic characterisation and epidemiology of 2019 novel coronavirus: implications for virus origins and receptor binding. Lancet. (2020) 395:565–74. doi: 10.1016/s0140-6736(20)30251-8, PMID: 32007145 PMC7159086

[3] Meyerowitz EA Richterman A Gandhi RT Sax PE. Transmission of SARS-CoV-2: a review of viral, host, and environmental factors. Ann Intern Med. (2021) 174:69–79. doi: 10.7326/m20-5008, PMID: 32941052 PMC7505025

[4] Abrahão JS Sacchetto L Rezende IM Rodrigues RAL Crispim APC Moura C. Detection of SARS-CoV-2 RNA on public surfaces in a densely populated urban area of Brazil: a potential tool for monitoring the circulation of infected patients. Sci Total Environ. (2021) 766:142645. doi: 10.1016/j.scitotenv.2020.142645, PMID: 33069469 PMC7530625

[5] van Doremalen N Bushmaker T Morris DH Holbrook MG Gamble A Williamson BN. Aerosol and surface stability of SARS-CoV-2 as compared with SARS-CoV-1. N Engl J Med. (2020) 382:1564–7. doi: 10.1056/NEJMc2004973, PMID: 32182409 PMC7121658

[6] Kampf G Todt D Pfaender S Steinmann E. Persistence of coronaviruses on inanimate surfaces and their inactivation with biocidal agents. J Hosp Infect. (2020) 104:246–51. doi: 10.1016/j.jhin.2020.01.022, PMID: 32035997 PMC7132493

[7] Han J Zhang X He S Jia P. Can the coronavirus disease be transmitted from food? A review of evidence, risks, policies and knowledge gaps. Environ Chem Lett. (2021) 19:5–16. doi: 10.1007/s10311-020-01101-x, PMID: 33024427 PMC7529092

[8] Liu P Yang M Zhao X Guo Y Wang L Zhang J. Cold-chain transportation in the frozen food industry may have caused a recurrence of COVID-19 cases in destination: successful isolation of SARS-CoV-2 virus from the imported frozen cod package surface. Biosaf Health. (2020) 2:199–201. doi: 10.1016/j.bsheal.2020.11.003, PMID: 33235990 PMC7676848

[9] COVID-19 Field Response Group, Laboratory Testing Group Song Y Zhao X Li X Xu W. A case of COVID-19—Tianjin municipality, China, 2020. China CDC Weekly. (2020) 2:884–5. doi: 10.46234/ccdcw2020.241, PMID: 34594791 PMC8393129

[10] Lin K Liu M Ma H Pan S Qiao H Gao H. Laboratory biosafety emergency management for SARS-CoV-2. J Biosaf Biosecur. (2020) 2:99–101. doi: 10.1016/j.jobb.2020.08.001, PMID: 32984781 PMC7501877

[11] Körner RW Majjouti M Alcazar MAA Mahabir E. Of mice and men: the coronavirus MHV and mouse models as a translational approach to understand SARS-CoV-2. Viruses. (2020) 12:880. doi: 10.3390/v12080880, PMID: 32806708 PMC7471983

[12] Ahmed W Bertsch PM Bibby K Haramoto E Hewitt J Huygens F. Decay of SARS-CoV-2 and surrogate murine hepatitis virus RNA in untreated wastewater to inform application in wastewater-based epidemiology. Environ Res. (2020) 191:110092. doi: 10.1016/j.envres.2020.110092, PMID: 32861728 PMC7451058

[13] Casanova L Rutala WA Weber DJ Sobsey MD. Survival of surrogate coronaviruses in water. Water Res. (2009) 43:1893–8. doi: 10.1016/j.watres.2009.02.002, PMID: 19246070 PMC7112071

[14] Bailey ES Curcic M Sobsey MD. Persistence of coronavirus surrogates on meat and fish products during long-term storage. Appl Environ Microbiol. (2022) 88:e0050422. doi: 10.1128/aem.00504-22, PMID: 35670583 PMC9238416

[15] Shen M Zhou Y Ye J Abdullah Al-Maskri AA Kang Y Zeng S. Recent advances and perspectives of nucleic acid detection for coronavirus. J Pharm Anal. (2020) 10:97–101. doi: 10.1016/j.jpha.2020.02.010, PMID: 32292623 PMC7102540

[16] Shao W Ye Q. SARS-CoV-2 spreads globally through the object-to-human transmission of cross-border logistics. Front Microbiol. (2022) 13:918957. doi: 10.3389/fmicb.2022.918957 35814665 PMC9260597

[17] Wang J Shen J Ye D Yan X Zhang Y Yang W. Disinfection technology of hospital wastes and wastewater: suggestions for disinfection strategy during coronavirus disease 2019 (COVID-19) pandemic in China. Environ Pollut. (2020) 262:114665. doi: 10.1016/j.envpol.2020.114665, PMID: 32443202 PMC7194566

[18] Wu X Chen Y Wang L Guo X Cui L Shen Y. Effectiveness of disinfectants suitable for inactivating SARS-CoV-2 at cold-chain temperature. Food Environ Virol. (2022) 14:101–4. doi: 10.1007/s12560-022-09509-0 35084667 PMC8792451

[19] Xiling G Yin C Ling W Xiaosong W Jingjing F Fang L. *In vitro* inactivation of SARS-CoV-2 by commonly used disinfection products and methods. Sci Rep. (2021) 11:2418. doi: 10.1038/s41598-021-82148-w, PMID: 33510320 PMC7843590

[20] Reed LJ Muench H. A simple method of estimating fifty per cent endpoints. Am J Epidemiol. (1938) 27:493–7. doi: 10.1093/oxfordjournals.aje.a118408

[21] Harbourt DE Haddow AD Piper AE Bloomfield H Kearney BJ Fetterer D. Modeling the stability of severe acute respiratory syndrome coronavirus 2 (SARS-CoV-2) on skin, currency, and clothing. PLoS Negl Trop Dis. (2020) 14:e0008831. doi: 10.1371/journal.pntd.0008831 33166294 PMC7676723

[22] Malenovská H. Coronavirus persistence on a plastic carrier under refrigeration conditions and its reduction using wet wiping technique, with respect to food safety. Food Environ Virol. (2020) 12:361–6. doi: 10.1007/s12560-020-09447-9, PMID: 33057921 PMC7557311

[23] Chatterjee S Murallidharan JS Agrawal A Bhardwaj R. Why coronavirus survives longer on impermeable than porous surfaces. Phys Fluids. (2021) 33:021701. doi: 10.1063/5.0037924 PMC7978145 33746485

[24] Ashokkumar S Kaushik NK Han I Uhm HS Park JS Cho GS. Persistence of coronavirus on surface materials and its control measures using nonthermal plasma and other agents. Int J Mol Sci. (2023) 24:14106. doi: 10.3390/ijms241814106, PMID: 37762409 PMC10531613

[25] Cox CS. Roles of water molecules in Bacteria and viruses. Orig Life Evol Biosph. (1993) 23:29–36. doi: 10.1007/bf01581988 11536524

[26] Fedorenko A Grinberg M Orevi T Kashtan N. Survival of the enveloped bacteriophage Phi6 (a surrogate for SARS-CoV-2) in evaporated saliva microdroplets deposited on glass surfaces. Sci Rep. (2020) 10:22419. doi: 10.1038/s41598-020-79625-z, PMID: 33376251 PMC7772334

[27] Guillier L Martin-Latil S Chaix E Thébault A Pavio N Le Poder S. Modeling the inactivation of viruses from the *Coronaviridae* family in response to temperature and relative humidity in suspensions or on surfaces. Appl Environ Microbiol. (2020) 86:e01244–20. doi: 10.1128/aem.01244-20, PMID: 32680860 PMC7480392

[28] Rutala W Weber DJ The Healthcare Infection Control Practices Advisory Committee, Guideline for disinfection and sterilization in healthcare facilities. (2008). Available at: https://www.cdc.gov/infectioncontrol/guidelines/disinfection/

[29] Xiao S Yuan Z Huang Y. Disinfectants against SARS-CoV-2: a review. Viruses. (2022) 14:1721. doi: 10.3390/v14081721 36016342 PMC9413547

[30] Miyaoka Y Kabir MH Hasan MA Yamaguchi M Shoham D Murakami H. Virucidal activity of slightly acidic hypochlorous acid water toward influenza virus and coronavirus with tests simulating practical usage. Virus Res. (2021) 297:198383. doi: 10.1016/j.virusres.2021.198383 33705798

[31] Salipante SJ Jerome KR. Digital PCR-an emerging technology with broad applications in microbiology. Clin Chem. (2020) 66:117–23. doi: 10.1373/clinchem.2019.304048, PMID: 31704712

[32] Seo M Lim H Park M Ha K Kwon S Shin J. Field study of the indoor environments for preventing the spread of the SARS-CoV-2 in Seoul. Indoor Air. (2022) 32:e12959. doi: 10.1111/ina.12959, PMID: 34806218 PMC9011577

[33] Wölfel R Corman VM Guggemos W Seilmaier M Zange S Müller MA. Virological assessment of hospitalized patients with COVID-2019. Nature. (2020) 581:465–9. doi: 10.1038/s41586-020-2196-x 32235945

[34] Harmooshi NN Shirbandi K Rahim F. Environmental concern regarding the effect of humidity and temperature on 2019-nCoV survival: fact or fiction. Environ Sci Pollut Res Int. (2020) 27:36027–36. doi: 10.1007/s11356-020-09733-w, PMID: 32592048 PMC7316637

