# Supplementary material for: The survival of murine hepatitis virus (a surrogate of SARS-CoV-2) on conventional packaging materials under cold chain conditions
Source: Front Public Health. 2023 Dec 5;11:1319828. doi: 10.3389/fpubh.2023.1319828 (PMC10728718; doi:10.3389/fpubh.2023.1319828)
Supplement: Supplementary file 1 [file Data_Sheet_1.docx]

**Supplementary Figure S1.** **The preparation of different packaging materials.**

From left to right, the materials are plastic, foam, cardboard, and wood sheets.


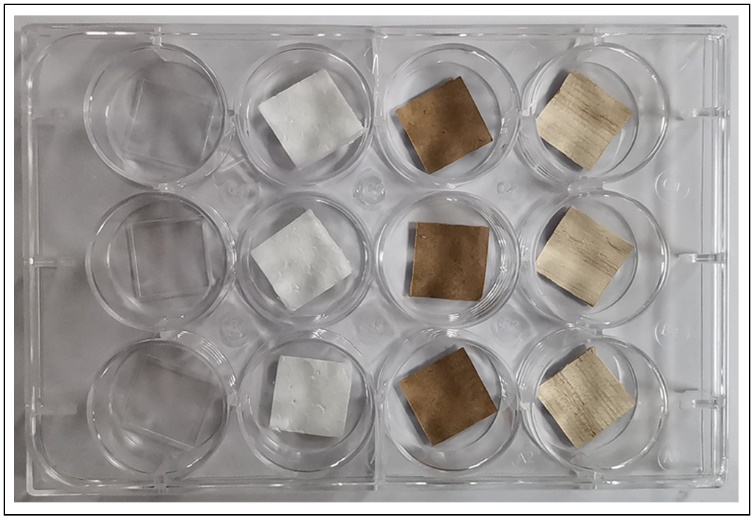


**Supplementary Figure S2. The primer validation results using PCR and qPCR.**

a. The electrophoresis image of the PCR amplification products. Lane 1 contains a 100 bp DNA ladder, and lanes 2 and 3 contain the PCR products. b. The fluorescence-based PCR melting curve.


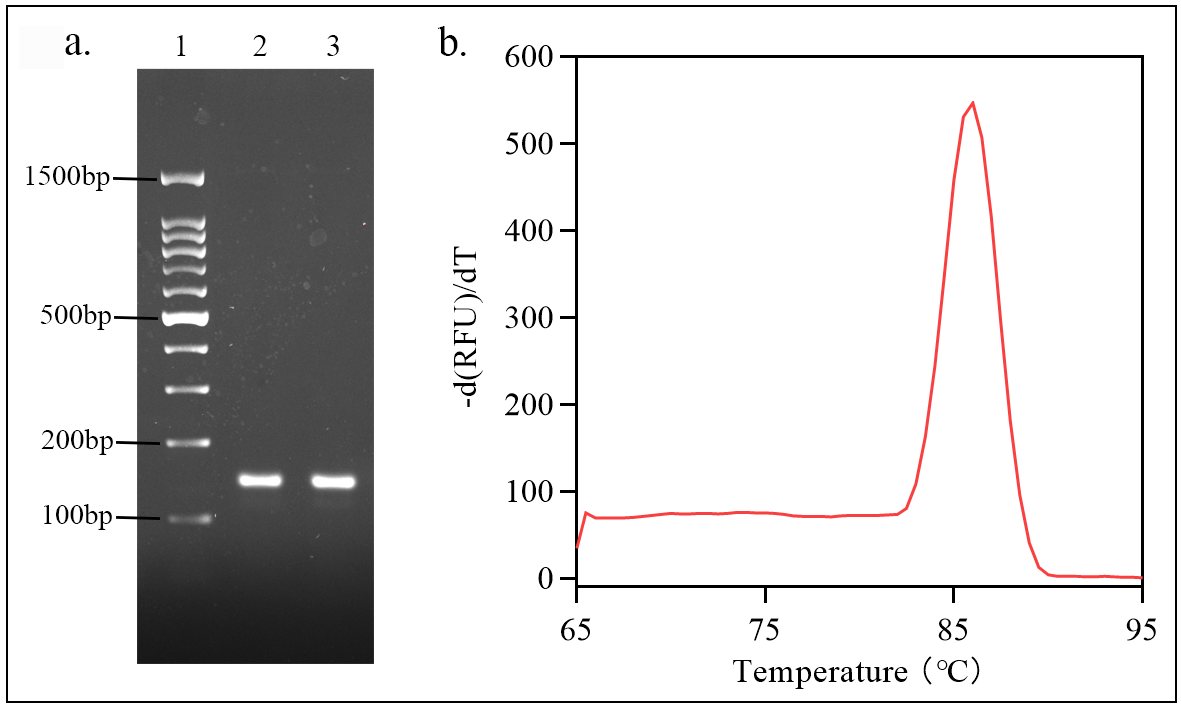


**Supplementary Figure S3. Histogram of dPCR analysis before optimizing probe concentration.**

a. Scatter plot of fluorescence signal intensities of positive and negative droplets. Fluorescence amplitude is plotted on the ordinate, and each droplet on the abscissa. Positive events are marked in blue, negative events in grey. b. Histogram of fluorescence signal intensities of positive and negative droplets. The horizontal and vertical axes represent fluorescence amplitude and droplet number, respectively. The fluorescence signal of positive droplets is on the right side of the boundary line. The threshold can be clearly set in between as indicated in the histogram.

**
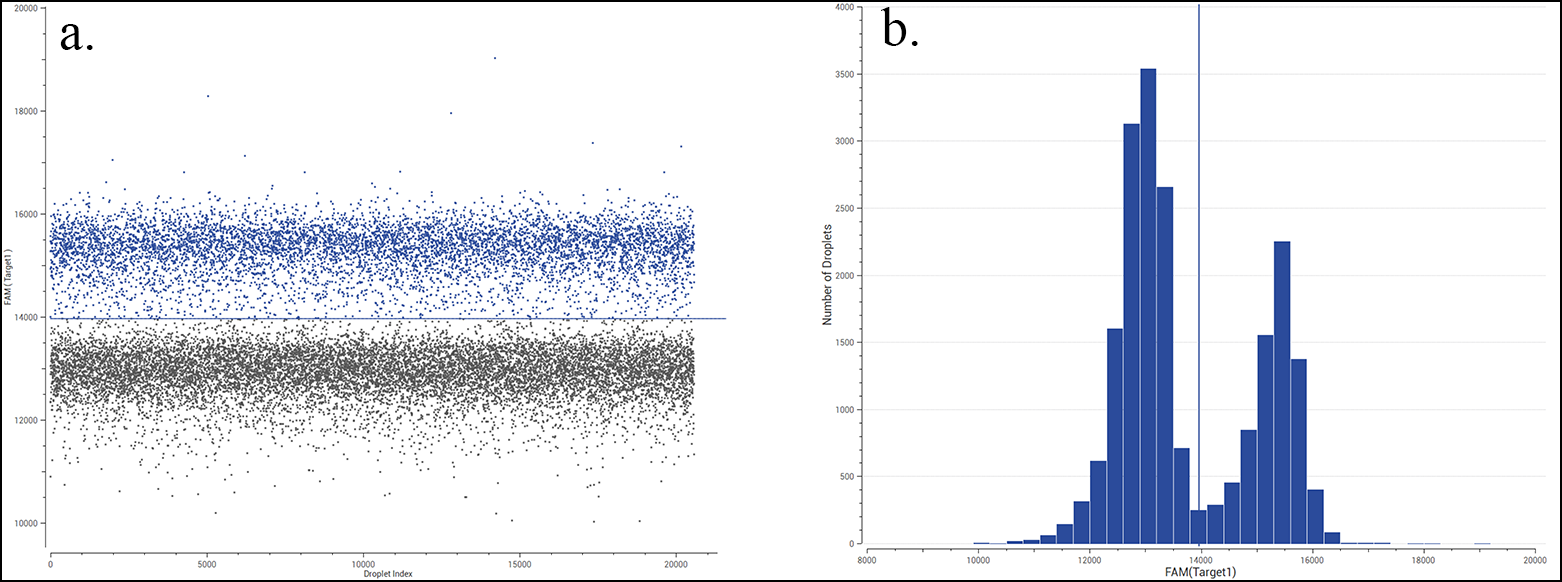
**

**Supplementary Figure S4. Histogram of dPCR analysis after optimizing probe concentration.**

a. Scatter plot of fluorescence signal intensities of positive and negative droplets. Fluorescence amplitude is plotted on the ordinate, and each droplet on the abscissa. Positive events are marked in blue, negative events in grey. b. Histogram of fluorescence signal intensities of positive and negative droplets. The horizontal and vertical axes represent fluorescence amplitude and droplet number, respectively. The fluorescence signal of positive droplets is on the right side of the boundary line. The threshold can be clearly set in between as indicated in the histogram.


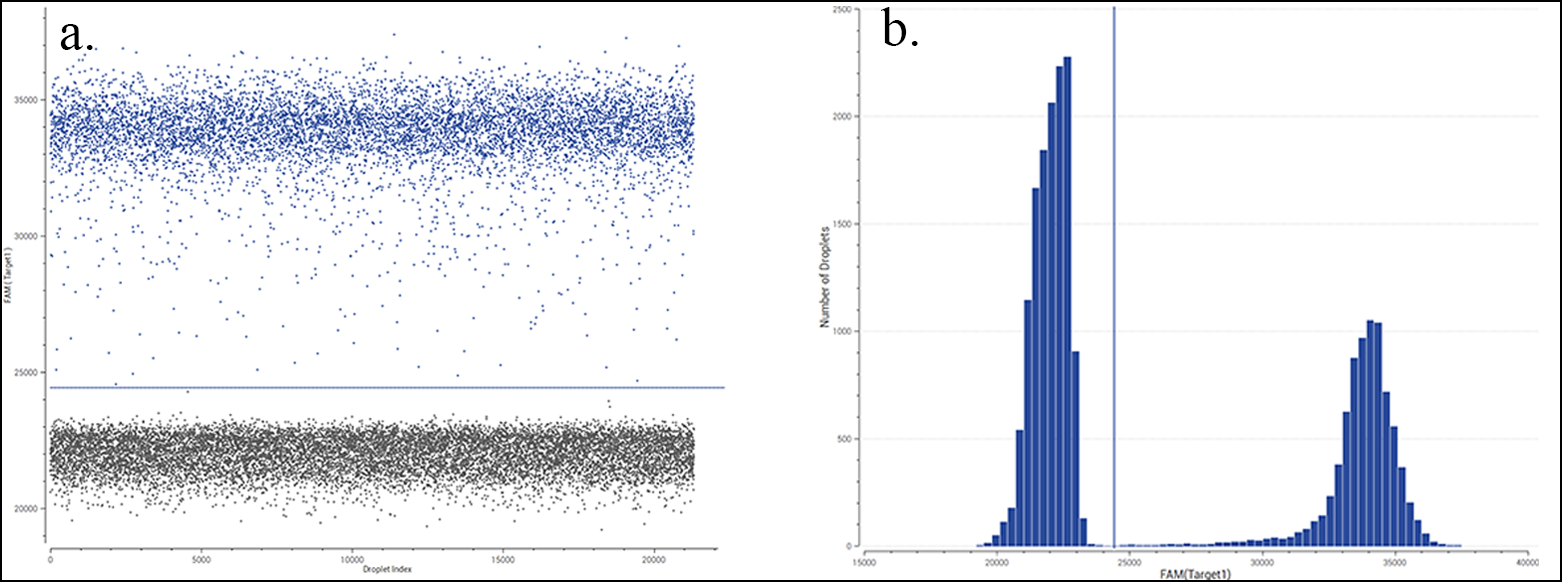


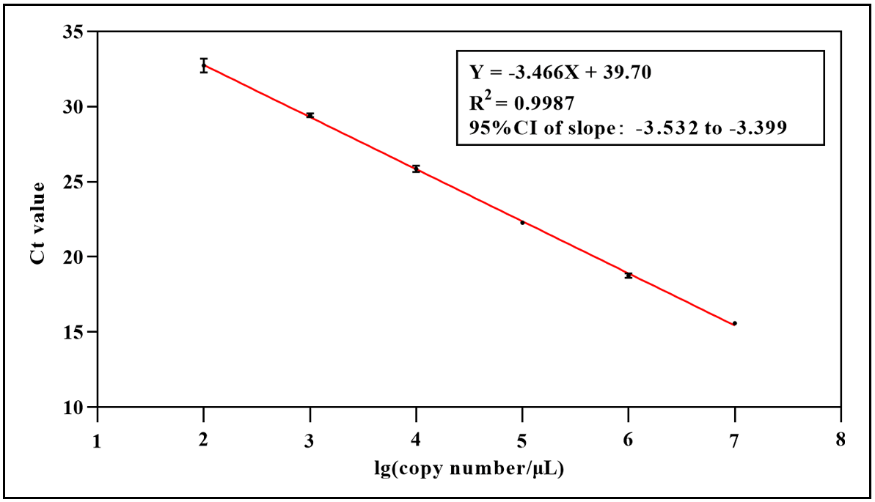


**Supplementary Figure S5. The linear regression equation between the Ct values and the logarithmically transformed copy numbers**

**Supplementary Table S1. The reaction components and steps of PCR and qPCR**

|  | Component | Volume (μL) | Step | Temperature (°C) | Time | Cycles |
| --- | --- | --- | --- | --- | --- | --- |
| PCR | T3 Super PCR Mix | 22 | Initial denaturation | 95 | 3 min | 1 |
|  | Forward primer | 1 | Denaturation | 95 | 10 s | 30 |
|  | Reverse primer | 1 | Annealling | 60 | 10 s |  |
|  | cDNA | 1 | Extension | 72 | 5 s |  |
|  |  |  | Final extension | 72 | 2 min | 1 |
|  |  |  |  |  |  |  |
| qPCR | SYBR Green qPCR Mix | 10 | Initial denaturation | 95 | 15 min | 1 |
|  | Forward primer | 0.6 | Denaturation | 95 | 10 s | 40 |
|  | Reverse primer | 0.6 | Annealling | 60 | 10 s |  |
|  | cDNA | 8.8 |  |  |  |  |

**Abbreviation:** PCR, polymerase chain reaction; qPCR, quantitative polymerase chain reaction; cDNA, complementary DNA.

**Supplementary Table S2.** **The components and steps of the reaction for detecting nucleic acid samples with known copy numbers using the Taqman probe-based qPCR**

| Component | Volume(μL) | Step | Temperature(°C) | Time | Cycles |
| --- | --- | --- | --- | --- | --- |
| 2×T5 Fast qPCR Mix（Probe） | 10 | Initial denaturation | 95 | 2 min | 1 |
| Forward primer | 0.8 | Denaturation | 95 | 10 s | 40 |
| Reverse primer | 0.8 | Annealling | 60 | 5 s |  |
| Taqman probe | 0.4 |  |  |  |  |
| Template | 2 |  |  |  |  |
| RNase free water | 6 |  |  |  |  |

**Abbreviation:** qPCR, quantitative polymerase chain reaction.

**Supplementary Table S3. Calculation of the dosage of disinfectant in spraying disinfection experiment.**

| Sample | Weight before spraying（g） | Weight after spraying（g） | Weight  difference（g） | Area  （cm^2^） | Weight per unit area（g/m^2^） |
| --- | --- | --- | --- | --- | --- |
| 1 | 1.79971 | 1.80280 | 0.00309 | 2.56 | 12.07031 |
| 2 | 1.79953 | 1.80783 | 0.00830 | 2.56 | 32.42187 |
| 3 | 1.80358 | 1.80868 | 0.00510 | 2.56 | 19.92188 |
| 4 | 1.80504 | 1.81083 | 0.00579 | 2.56 | 22.61719 |
| 5 | 1.80116 | 1.80773 | 0.00657 | 2.56 | 25.66406 |
| 6 | 1.80508 | 1.80919 | 0.00411 | 2.56 | 16.05469 |
| mean | 1.80235 | 1.80784 | 0.00549 | 2.56 | 21.45833 |
